# Rapid Bidirectional Reorganization of Cortical Microcircuits

**DOI:** 10.1093/cercor/bhu098

**Published:** 2014-05-16

**Authors:** Giorgia Albieri, Samuel J. Barnes, Benito de Celis Alonso, Claire E.J. Cheetham, Clarissa E. Edwards, Andrew S. Lowe, Harini Karunaratne, John P. Dear, Kalok C. Lee, Gerald T. Finnerty

**Affiliations:** 1 MRC Centre for Neurodegeneration Research, King's College London, Institute of Psychiatry (Box44), London SE5 8AF, UK; 2 Department of Mechanical Engineering, Imperial College London, London SW7 2AZ, UK; 3 Division of Engineering, King's College London, Strand, London WC2R 2LS, UK; 4 Current address: Division of Neurobiology, MRC Laboratory of Molecular Biology, Cambridge CB2 0QH, UK; 5 Current address: MRC Centre for Developmental Neurobiology King's College London, Guy's Hospital Campus, London SE1 1UL, UK; 6 Current address: Faculty of Physics and Mathematics, prior to the University, Benemérita Universidad Autónoma de Puebla, Puebla, Mexico; 7 Current address: National Institutes of Health, Bethesda, MD, USA

**Keywords:** connectome, cortical microcircuit, experience-dependent plasticity, fMRI, inhibition, rewiring

## Abstract

Mature neocortex adapts to altered sensory input by changing neural activity in cortical circuits. The underlying cellular mechanisms remain unclear. We used blood oxygen level-dependent (BOLD) functional magnetic resonance imaging (fMRI) to show reorganization in somatosensory cortex elicited by altered whisker sensory input. We found that there was rapid expansion followed by retraction of whisker cortical maps. The cellular basis for the reorganization in primary somatosensory cortex was investigated with paired electrophysiological recordings in the periphery of the expanded whisker representation. During map expansion, the chance of finding a monosynaptic connection between pairs of pyramidal neurons increased 3-fold. Despite the rapid increase in local excitatory connectivity, the average strength and synaptic dynamics did not change, which suggests that new excitatory connections rapidly acquire the properties of established excitatory connections. During map retraction, entire excitatory connections between pyramidal neurons were lost. In contrast, connectivity between pyramidal neurons and fast spiking interneurons was unchanged. Hence, the changes in local excitatory connectivity did not occur in all circuits involving pyramidal neurons. Our data show that pyramidal neurons are recruited to and eliminated from local excitatory networks over days. These findings suggest that the local excitatory connectome is dynamic in mature neocortex.

## Introduction

Behavioral experience, learning, and memory result in reorganization of neural circuitry in the brain (Martin et al. 2000; Gilbert and Li 2012). Studies of the reorganization at a cellular level have focused on 3 broad classes of plasticity mechanism: Changes in synaptic strength, altered excitability of neurons, and rewiring of neural circuits (Martin et al. 2000; Zhang and Linden 2003; Feldman 2009; Barnes and Finnerty 2010). However, despite intensive investigation, we have limited understanding of how cellular plasticity mechanisms enable the neocortex to reorganize in response to sensory and motor experience.

Unraveling the cellular plasticity mechanisms that cause reorganization of cortical maps has been difficult because the findings have varied with the experimental protocol. However, cortical reorganization induced by nontraumatic alterations to sensory experience and by training on a task are thought to be mechanistically similar (Gilbert and Li 2012). Early mapping studies in adult neocortex suggested that the representations of the trained digits in somatosensory cortex (Recanzone et al. 1992) or trained tones in auditory cortex (Recanzone et al. 1993) were expanded. However, several later studies of perceptual and motor learning indicate that cortical map expansion may not be persistent. Instead, cortical maps may expand during learning, but then return to baseline levels after the task has been learnt (Molina-Luna et al. 2008; Yotsumoto et al. 2008; Reed et al. 2011; Gilbert and Li 2012). The latter view suggests that map expansion and contraction is a period of intense cortical reorganization.

In rodent somatosensory cortex, plasticity mechanisms have commonly been investigated after altering the sensory input from a rodent's snout, for example, by trimming a subset of its whiskers. Neural firing in primary somatosensory cortex (SI) that has lost its principal whisker sensory input (deprived cortex) has been reported to be adjusted in at least 2 ways. Recordings from microelectrodes inserted into SI have revealed that stimulation of the remaining, intact whiskers evokes more firing in the upper layers of deprived cortex (Diamond et al. 1994; Glazewski and Fox 1996). However, microelectrodes typically capture the firing of only a few active neurons. A distinct insight has emerged from studies that follow populations of active and inactive neurons in SI with in vivo calcium imaging (Margolis et al. 2012). After whisker trimming, the firing of layer 2/3 (L2/3) pyramidal neurons in deprived cortex is redistributed. Specifically, neurons that responded poorly to whisker stimulation in the naive rodent fire more action potentials, whereas those that responded reliably in the naive rodent fire less action potentials (Margolis et al. 2012). This finding emphasizes the role of recruiting previously inactive, “silent” neurons (Shoham et al. 2006) into local excitatory networks during cortical plasticity. The mechanism whereby cortical microcircuits recruit new neurons is currently unknown.

Disinhibition is widely thought to play a role in reorganization of the adult neocortex (Jacobs and Donoghue 1991; Jones 1993; Chen et al. 2011; Keck et al. 2011; van Versendaal et al. 2012) and may be involved in expanding excitatory networks. This may occur through a finite period (days) of weakening of inhibitory circuitry, which reveals latent excitatory connections (Jacobs and Donoghue 1991) or facilitates plasticity of excitatory circuitry (Chen et al. 2011; Keck et al. 2011; van Versendaal et al. 2012). Rapid structural changes to inhibitory circuits occur in L2/3 of deprived cortex and are consistent with the disinhibition hypothesis (Marik et al. 2010; Chen et al. 2011; Keck et al. 2011; van Versendaal et al. 2012). However, there is little direct, functional evidence for disinhibition in L2/3 of deprived cortex, where plasticity is greatest.

We investigated the mechanisms underlying adult cortical reorganization by combining blood oxygen level-dependent (BOLD) functional magnetic resonance imaging (fMRI) to image whisker cortical maps with electrophysiological recordings from pairs of synaptically connected neurons. We show that cortical map expansion is accompanied by a rapid increase in the connectivity between L2/3 pyramidal neurons in the periphery of the expanded map. Map retraction is associated with the loss of entire local excitatory connections. The rewiring does not affect local inhibitory circuits. We propose that the rewiring reconfigures local excitatory circuits.

## Materials and Methods

### Whisker Trimming

All procedures were carried out in accordance with the UK Animals (Scientific Procedures) Act 1986. For BOLD fMRI experiments, all whiskers except for the C row whiskers bilaterally of adult Sprague-Dawley rats (250–350 g) were cut daily to the level of the facial hair. This trimming protocol generates multiple boundaries between spared and deprived cortex in SI at the junction of: (1) The C and D barrel columns (medially); (2) the C and B barrel columns (laterally); and (3) the β and γ straddler whiskers caudally. Control rats were sham-trimmed. All rats' whiskers except for the C1–4 whiskers bilaterally were cut immediately prior to scanning. For electrophysiology experiments, we trimmed the lower 2 rows (D and E rows, γ and δ outliers) of rats' whiskers to reproduce the imaging-experiment boundary between deprived and spared cortex at the junction of C and D barrel columns. Trimming was performed daily from postnatal day 30 (P30) for 2–4 days or 6–8 days.

### MRI and fMRI Methods

Our imaging protocol has been previously described (Alonso et al. 2008). Male Sprague-Dawley rats were anesthetized with α-chloralose. Imaging was performed in a horizontal bore 9.4 T magnet with a 25-mm diameter surface coil. The right C1–4 whiskers were moved rostro-caudally at 5 Hz by a pneumatic system. An imaging session consisted of 120 blocks with 60 blocks ON (whisker deflection throughout block) and 60 blocks OFF (no whisker deflection). The volume of data acquired in each block comprised 12 slices of 0.5 mm thickness. The 0.5-mm slice thickness approximates to the diameter of one barrel column in SI (Riddle and Purves 1995). A multiecho gradient echo (GE) imaging sequence was custom-written to improve the contrast to noise of the BOLD signal. Imaging parameters were: flip angle, 31°; time repetition (TR) = 340 ms; time echo (TE) = 4, 8, 12, 16, 20 ms; field of view (FOV), 32 × 32 mm; matrix, 96 × 96. The acquisition time per volume was 32.6 s and the total scan time per fMRI session was 1 h 5 min. The ON and OFF blocks were randomized to reduce colored noise attributable to vascular pulsations accompanying the cardiac and respiratory cycles with the conditions that: (1) The protocol always begins with an OFF and (2) a maximum of 2 ONs occur in succession (Alonso et al. 2008). Coronal anatomical scans were created from a spin-echo sequence (TR/TE = 1000/20 ms), with 0.5 mm thick slices, a FOV of 32 × 32 mm, a matrix size of 192 × 192, and 4 signals averaged.

### fMRI Data Analysis

Multiecho GE data sets were converted into single-echo images (effective TE = 12 ms) by summing the magnitude images generated from each echo. Images from each scan session were co-registered and aligned in SPM99 (http://www.fil.ion.ucl.ac.uk/spm/). Any rat that exhibited head motion exceeding 0.5 mm along *x* or *y* axes or >0.75 mm in the *z* direction (along B_0_) was excluded from further analysis. We minimized BOLD signal attributable to large draining veins and vascular inflow (Menon and Goodyear 2001) by constructing a coefficient of variation map of the BOLD signal and eliminating voxels with coefficients of variation greater than 15% (Hlustik et al. 1998). We reduced noise in our functional images by performing a probabilistic independent component analysis on 4D data sets using MELODIC 2.0 (http://www.fmrib.ox.ac.uk/fsl/). Components that had a correlation coefficient with a *P-*value of >0.1 when compared with the stimulus paradigm, that is, uncorrelated, were removed by linear regression to form a denoised data set. Data were smoothed with a Gaussian kernel (full width half maximum, 0.99 mm). A general linear model of the data was constructed (Alonso et al. 2008). A BOLD response was deemed present in single-animal statistical parametric maps if there was a cluster of 4 or more contiguous voxels that were all statistically significant (*P* < 0.05, uncorrected) in the region of interest (Alonso et al. 2008).

### Brain Slice Preparation and Electrophysiological Recording

Brain slices were cut across the whisker barrel rows (Cheetham et al. 2007). We made whole-cell voltage recordings of synaptically connected pairs of L2/3 pyramidal neurons in spared and control cortex at 36–37 °C. Recording pipettes (4–7 MΩ) for voltage recordings contained (in mM): KMeSO_4_ 130, NaCl 8, KH_2_PO_4_ 2, d-Glucose 2, HEPES 10, MgATP 4, GTP 0.3, ADP K Salt 0.5, Alexa Fluor 488 (AF488) 1 or Alexa Fluor 568 (AF568) 1 (Invitrogen, UK), and biocytin 3 mg/mL. Miniature excitatory postsynaptic potentials (mEPSPs) and unitary EPSPs (uEPSPs) were recorded and analyzed as described previously (Cheetham et al. 2007). Probability of failure was calculated from responses to the first action potential in the stimulus train. Neuronal excitability was investigated by injecting 500 ms current pulses into the soma to evoke action potential firing. Connectivity between control neurons and uEPSP amplitude did not change between the P32–P34 and P36–38 groups and was pooled. uPSP responses (uEPSP or unitary inhibitory postsynaptic potential (uIPSP)) were normalized to the first response (uPSP1) in the train. The normalized steady-state amplitude in the train was the average of the sixth to eighth responses (uEPSP6–8) in the train after normalization.

Miniature inhibitory postsynaptic currents (mIPSCs) were recorded from pyramidal neurons in voltage clamp, with the resting membrane potential held at 0 mV. The internal solution contained (in mM): Cesium methanesulfonate (CH_3_O_3_SCs) 130, NaCl 8, KH_2_PO_4_ 2, Dextrose 2, HEPES 10, MgATP 4, GTP 0.3, ADP K Salt 0.5, QX-314 bromide 10, either Alexa Fluor (AF) 488 1 or AF 568 1 (Invitrogen, UK), and biocytin 3 mg/mL. Pyramidal cells were excluded if *V*_m_ at break in was >−68 mV, *R*_s_ > 35 MΩ, or *R*_m_ < 100 MΩ. uIPSPs were recording in current clamp with the same internal solution that was used for pyramidal neurons. The resting membrane potential of the L2/3 pyramidal neuron was held at −55 mV to increase the amplitude of the uIPSP. Fast spiking (FS) interneurons were identified based on morphological characteristics. Their identity was then confirmed electrophysiologically, by analyzing the firing response to 500 ms pulses of depolarizing currents (0.4–1.0 nA). Interneurons were considered FS if they reached firing frequencies >200 Hz in response to 1.0 nA current injection. FS interneurons were excluded if they had bouton cartridges aligned along the axis of the barrel column in fluorescence images, suggesting that they were axo-axonic cells (Supplementary Fig. 1).

### Confocal Imaging and Dendritic Spine Counts

Confocal laser-scanning microscopy and spine counts were performed as described previously (Cheetham et al. 2007, 2008). Briefly, confocal images were acquired with a Zeiss 510 META confocal microscope with a C-Apochromat 63× water-immersion objective and imaged with Imaris (Bitplane). Spine densities were measured by scrolling up and down through a dendrite and counting all spines in a 10-μm section at a measured path length from the soma.

### Statistics

Normally distributed data were described by their mean ± SEM and were analyzed using *t*-tests or analysis of variance (ANOVA). Data that failed normality and/or equal variance tests were expressed as median [interquartile range]. Where possible, these data either underwent a natural log transform to normalize their distributions and/or equalize their variances prior to performing *t*-tests or ANOVA. The Mann–Whitney rank sum test was used to compare medians. Distributions were compared using the Kolmogorov–Smirnov test (Cheetham et al. 2007) (Matlab, Mathworks). The volumes of SI positive BOLD responses (PBRs) were analyzed with Poisson regression in R (R Project for Statistical Computing, http://www.r-project.org/) using the quasi family to enable modeling of overdispersion and the formula:

log[E(SI PBR volume)]=α+β(peak PBR amplitude)+γ(3-day trim)+δ(7-day trim),

where *E*(SI PBR volume) is the expected value of the SI PBR volume, “3-day trim” and “7-day trim” are dummy variables, and *α*, *β*, *γ*, and *δ* are parameters (coefficients) of the model. Spine densities were analyzed with a general additive model using the “mgcv” and “gam” packages in R and the formula:

E(spine density)=α+s(path length from soma)+β(DEP),

where *E*(spine density) is the expected spine density, *s*(path length from soma) is a smoothed function of the distance along the dendrite of the spines from the soma, DEP is a dummy variable (1, deprived; 0, control), and *α* and *β* are parameters (coefficients) of the model.

## Results

### Expansion of Whisker Representations Imaged with fMRI

**Figure 1.**
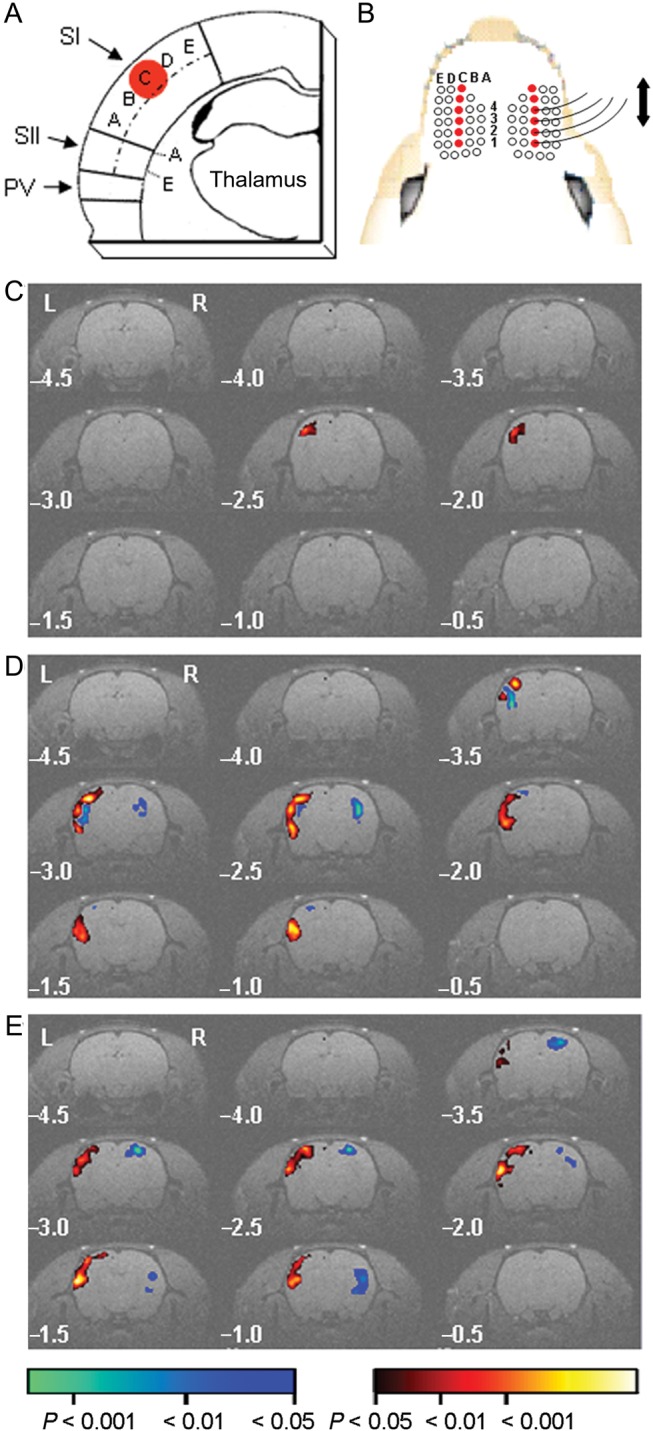
Spared whisker representations enlarge after whisker trimming. (*A*) Schematic illustrating the relative position of SI, SII, and the parietal ventral area (PV) in a coronal slice through whisker barrel cortex. Dashed line bisects SI and SII. Whisker barrel columns are marked *A*–*E*. Red circle, SI PBR evoked by whisker deflection. (*B*) Schematic of trimming protocol (open circle denotes trimmed whisker) and deflection of right C1–4 whiskers. Left C1–4 whiskers not shown. (*C* and *E*) Group statistical parametric maps of BOLD responses evoked by 5 Hz whisker deflection in sham-trimmed rats (*C*, *n* = 26 rats), and after whisker trimming for 3 days (*D*, *n* = 15 rats) and 7 days (*E*, *n* = 28 rats). Pseudocolored voxels have a positive (red) or negative (blue) BOLD signal that is significantly different from baseline. Pseudocolor scale bar applies to (*C* and *E*). Numbers indicate rostro-caudal distance from bregma.

**Figure 2.**
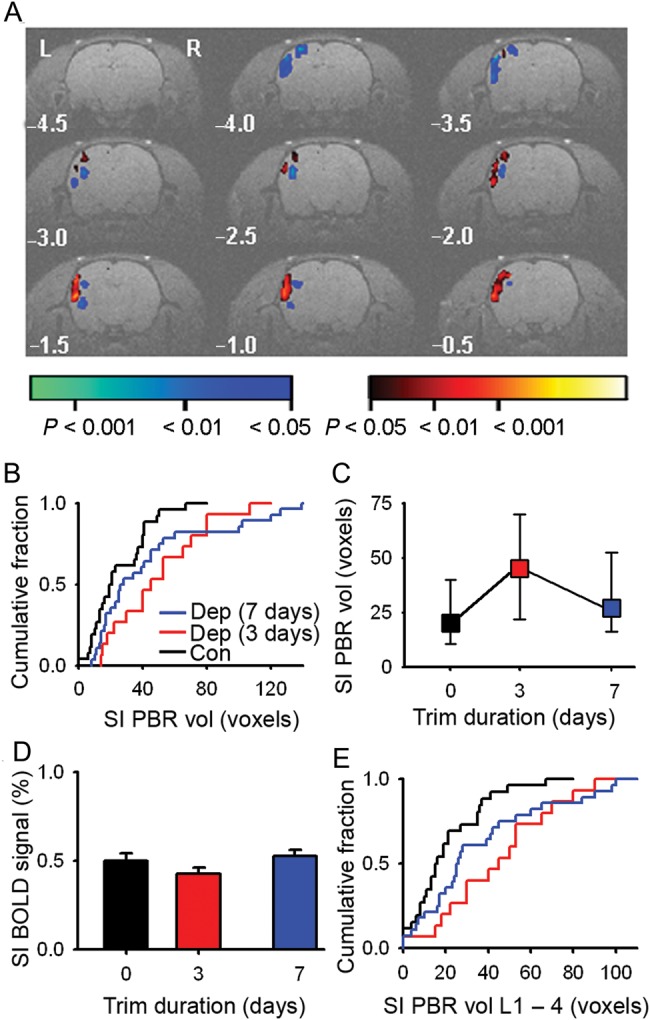
SI whisker representation expands without an increase in BOLD signal amplitude. (*A*) Single-animal statistical parametric map of the BOLD signal evoked by 5 Hz deflection of the right C1–4 whiskers after 3 days of whisker trimming. (*B*) Cumulative fraction plot of SI PBR volume from single-animal maps for controls (black, *n* = 26 rats), 3-day trim (red, *n* = 15 rats), and 7-day trim (blue, *n* = 28 rats). (*C*) Median SI PBR volume and interquartile range (error bars) after 3 and 7 days of whisker trimming (median SI PBR volume: controls, 20 [11–40] voxels, *n* = 26 rats; 3-day trim, 45 [24–69] voxels; *n* = 15 rats; 7-day trim, median volume, 26 [17–51] voxels, *n* = 28 rats). (*D*) Peak amplitude of BOLD signal (error bars, SEM) after whisker trimming (control, +0.50 ± 0.04%, *n* = 26 rats; 3-day trim, +0.43 ± 0.04%, *n* = 15 rats; 7-day trim, +0.53 ± 0.04%, *n* = 28 rats). (*E*) Cumulative fraction plot of the SI PBR volume in L1–4. Color code for (*C*–*E*) as (*B*).

Early processing of touch sensory information in rodent neocortex occurs in distinct maps that lie in SI and secondary somatosensory cortex (SII) with a third rudimentary map in the parietal ventral area (Chapin and Lin 1984; Benison et al. 2007) (Fig. 1*A*). Experience-dependent plasticity of whisker maps was induced in adult somatosensory cortex by daily bilateral trimming of all whiskers except for the C row (Fig. 1*B*). This protocol facilitates detection of plasticity in SI because it enables the representation of spared whiskers to expand in multiple directions. We simulated normal whisking in the MRI scanner by passively deflecting the right-sided C1–C4 whiskers at 5 Hz and used the evoked BOLD signal as a read-out of reorganization of whisker cortical maps (Fig. 1*B*). In control rats, synchronous deflection of the C1–C4 whiskers evoked a PBR in contralateral SI that extended over 2 imaging slices in the group statistical map (Fig. 1*C*). In contrast, deflection of the spared whiskers after trimming for either 3 days (Fig. 1*D*; Supplementary Material and Fig. 2) or 7 days (Fig. 1*E*) elicited PBRs in the grouped data that extended over 6 contiguous slices in contralateral SI, SII, and the parietal ventral area. The grouped data also showed negative BOLD responses in contralateral somatosensory cortex after 3 days (Fig. 1*D*), but not after 7 days of trimming (Fig. 1*E*).

The cortical representation of rats' whiskers may show marked variability in mapping studies (Riddle and Purves 1995; Chen-Bee and Frostig 1996). This has a direct impact on how best to quantify cortical reorganization with BOLD fMRI (Woods 1996; Petersson et al. 1999a, 1999b; Thirion et al. 2007). Group maps derived from a fixed effects model show small-amplitude changes in the BOLD signal. However, the group maps do not allow for interanimal variability in the location of the whisker map evident in single-animal data sets (Supplementary Fig. 2) and tend to give greater weight to animals with larger PBRs. Accordingly, we quantified the changes in the PBR in SI using single-animal statistical maps (Fig. 2*A*) (Alonso et al. 2008). SI PBR volume increased markedly after whisker trimming for 3 days (Fig. 2*B*,*C*) (median [interquartile range]: 3-day trim, 45 [24–69] voxels, *n* = 15 rats; controls, 20 [11–40] voxels, *n* = 26 rats; *P* < 0.001, Methods). Spared SI whisker representations had shrunk back after 7 days of whisker trimming (vs. 3-day trim volume, *P* = 0.008), but remained larger than control representations (7-day trim, 26 [17–51] voxels, *n* = 28 rats, *P* = 0.004; Fig. 2*B*,*C*). In contrast, the amplitude of the BOLD signal in contralateral SI after either 3 or 7 days of whisker trimming was similar to control values (Fig. 2*D*) (control, +0.50 ± 0.04%, *n* = 26 rats; 3-day trim, +0.43 ± 0.04%, *n* = 15 rats; 7-day trim, +0.53 ± 0.04%, *n* = 28 rats; *P* = 0.261, one-way ANOVA). We concluded that our BOLD imaging showed rapid reorganization, mainly at the periphery of spared whisker representations in SI.

Whisker map plasticity is greatest in the supragranular layers of somatosensory cortex (Diamond et al. 1994). Therefore, we determined whether the expansion of the PBR was uniform throughout the SI neocortex by dividing it into upper and lower halves (Alonso et al. 2008), which are congruent with layers 1–4 (L1–4) and layers 5–6, respectively. The volume of the PBR in L1–4 increased after 3 days (*P* < 0.001) of whisker trimming (Fig. 2*E* and Supplementary Material). The map retracted between 3 and 7 days of whisker trimming (3- vs. 7-day, *P* = 0.004), but had not shrunk back to control dimensions (7-day vs. control, *P* = 0.004). The increase in volume of the L1–4 PBR accounted for the majority of the expanded PBR (Fig. 2*B*,*E*). Hence, our BOLD imaging is consistent with evolving reorganization in upper cortical layers.

Changes in BOLD signal were not confined to SI whisker representations. A PBR was elicited in SII after 3 days (Fig. 2*A*) and after 7 days of whisker trimming, but not in control animals (SII PBR amplitude and volume: 3-day trim, +0.35% [0.24–0.43%], 19 [6–40] voxels, *n* = 15 rats; 7-day trim, +0.34% [0.00–0.46%], 8 [0–33] voxels, *n* = 28 rats; control, +0.00% [0.00–0.37%], 0 [0–18] voxels, *n* = 26 rats). A multifocal negative BOLD response was adjacent to the PBRs in somatosensory cortex (total negative BOLD volume: 3-day trim 33 [15–51] voxels; 7-day trim 26 [0–38] voxels; control 0 [0–29] voxels). We concluded that our imaging data showed plasticity of multiple whisker cortical maps that evolved over days.

### Local Excitatory Circuits Rewire in the Periphery of the Expanded Whisker Representation

**Figure 3.**
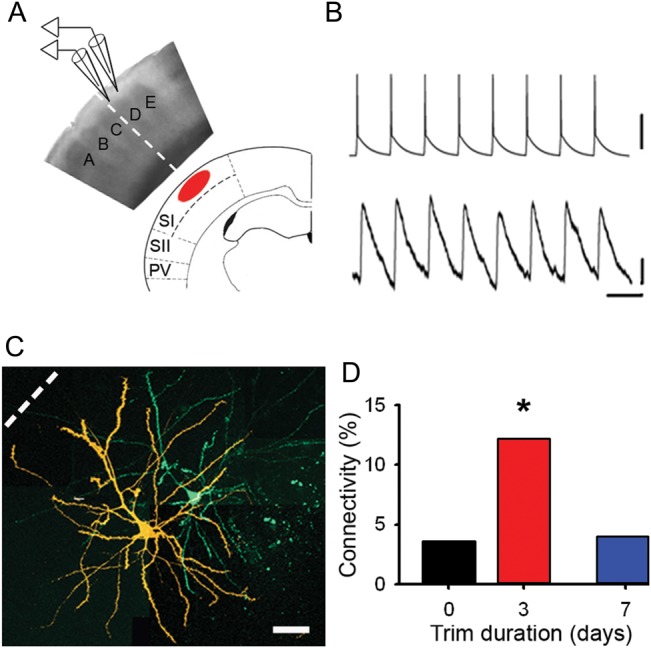
Local excitatory connectivity changes in concert with BOLD whisker representations. (*A*) Schematic showing orientation of a brain slice with respect to BOLD fMRI images. Electrophysiological recordings were made in L2/3. Dashed line indicates the boundary between spared C-row whiskers and deprived cortex. (*B*) Synaptically connected pyramidal neurons. Upper trace, train of action potentials in presynaptic neuron. Lower trace, evoked response in postsynaptic neuron. Scale bars: 50 mV (upper), 0.1 mV (lower); 50 ms. (*C*) Confocal reconstruction of the presynaptic (green) and postsynaptic (orange) pyramidal neurons. Scale bar, 50 μm. (*D*) Connectivity between deprived L2/3 pyramidal neurons in controls (black, 3.6%), after whisker trimming for 2–4 days (red, 12.0%) and after whisker trimming for 6–8 days (blue, 4.0%).

We next investigated the cellular basis for the cortical reorganization. We made electrophysiological recordings in SI that had been deprived of its principal whisker sensory input. Recordings were focused on L2/3 of deprived cortex adjacent to spared cortex because this region lies in the periphery of the expanded PBR where our functional imaging indicated that reorganization was occurring. We prepared brain slices that cut across the whisker barrel rows and made recordings from pairs of pyramidal neurons in L2/3 near the junction between the C and D barrel columns (Cheetham et al. 2007, 2008) (Fig. 3*A*–*C*), where spared representations had expanded into deprived cortex. In control cortex, the chance of finding a connection between neighboring L2/3 pyramidal neurons (Pyr → Pyr) was low (21/553 is 3.8%, 21/553 tested Pyr → Pyr pairs). In contrast, there was a dramatic increase (>3-fold) in Pyr → Pyr connectivity in deprived cortex after 2–4 days of whisker trimming (12.0%, 16/133 connections tested, *P* < 0.001, *χ*^2^ test) (Fig. 3*D*). After trimming for 6–8 days, connectivity in deprived cortex had returned to control levels (4.0%, 8/201 connections tested, *P* = 0.988, *χ*^2^ test) (Fig. 3*D*). Hence, the reorganization of local excitatory circuitry follows the same temporal pattern as the expansion and retraction of the spared whisker representations imaged with BOLD fMRI.

**Figure 4.**
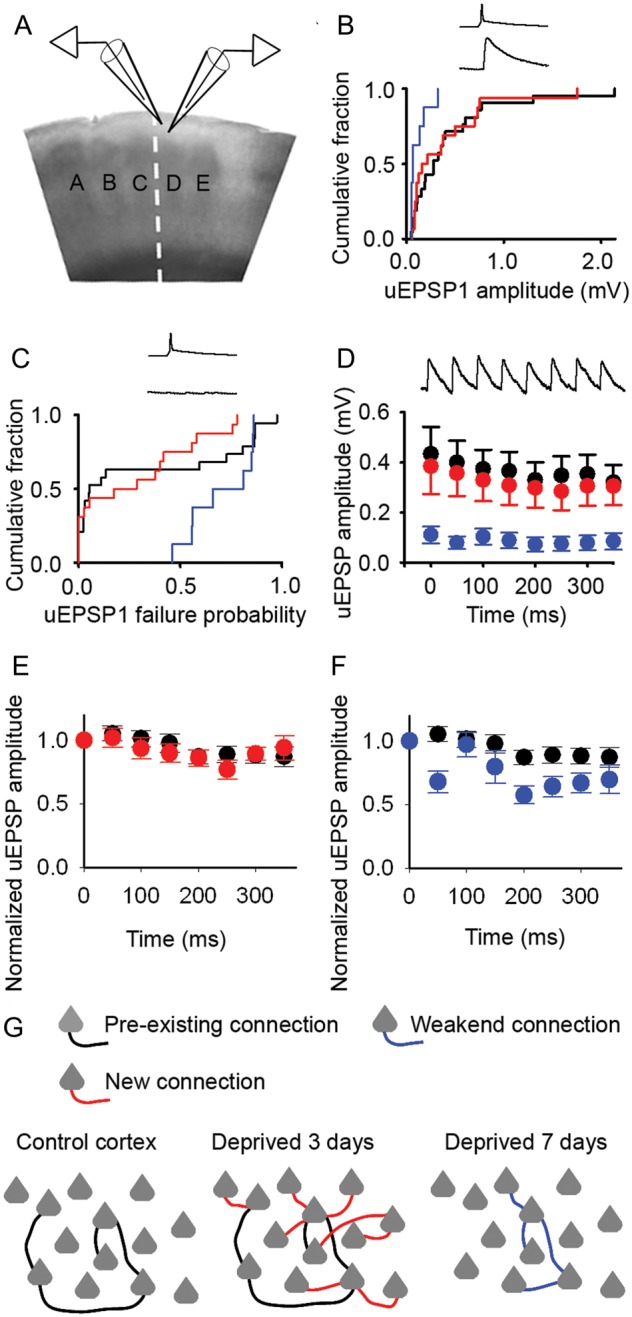
New excitatory connections in deprived cortex have similar properties to control connections. (*A*) Schematic of recordings from pairs of synaptically connected L2/3 pyramidal neurons. (*B*) Mean uEPSP amplitudes in control cortex (black) and deprived cortex after 2–4 days (red) or 6–8 days (blue) of whisker trimming (2- to 4-day trim, 0.38 ± 0.11 mV, *n* = 16; 6- to 8-day trim, 0.11 ± 0.03 mV, *n* = 8; control, 0.43 ± 0.11 mV, *n* = 21). Inset: presynaptic action potential, postsynaptic EPSP. (*C*) Failure rates of neurotransmission between pyramidal neurons after 2–4 days (red) and 6–8 days (blue) of whisker trimming (median [IQR]: 2- to 4-day trim, 0.23 [0.00–0.49], *n* = 16; 6- to 8-day trim, 0.74 [0.56–0.85], *n* = 8; control, 0.05 [0.02–0.79], *n* = 19). Inset: presynaptic action potential, no postsynaptic response. (*D*) uEPSP amplitudes during a 20-Hz stimulus train in control cortex (filled circles) or deprived cortex after 2–4 days (red) and 6–8 days (blue) of whisker trimming. Error bars, SEM. Inset: 20 Hz train of postsynaptic EPSPs. (*E*) Normalized uEPSP amplitudes of Pyr → Pyr connections during a 20-Hz train in deprived cortex after 2–4 days trimming (red, *n* = 16) and in control cortex (black, *n* = 21). Error bars, SEM. (*F*) Normalized uEPSP amplitudes of Pyr → Pyr connections during a 20-Hz train in deprived cortex after 6–8 days trimming (blue, *n* = 8) and in control cortex (black, *n* = 21). Error bars, SEM. (*G*) Schematic shows sparse local connectivity in control cortex prior to whisker trimming (left panel). Whisker trimming for 3 days induces a rapid 3-fold increase in local excitatory connectivity (middle panel; new connections, red). Connectivity returns to control levels following 7 days of whisker trimming (right panel; 7-day trim connections, blue).

Local excitatory connections are not random, but form microcircuits (Yoshimura et al. 2005; Cheetham et al. 2008). Typically, an excitatory connection between 2 pyramidal neurons in a microcircuit is formed by multiple synapses (Cheetham et al. 2007). We reasoned that the formation of new local excitatory connections in deprived cortex might lead to increased synapse number in deprived cortex. We addressed this issue in 2 ways. We counted dendritic spines on L2/3 pyramidal neurons to give a structural measure of synapse number. Secondly, we recorded mEPSPs to give a functional measure of total excitatory synaptic drive onto pyramidal neurons. We found no change in either spine density (Supplementary Fig. 3) or mEPSP amplitude and frequency (Supplementary Material and Fig. 4). The relative stability of the total number of excitatory synapses indicates that the formation of new local excitatory connections was offset by loss of synaptic inputs from other pathways. This may be mediated by synapse turnover, which is increased by whisker deprivation (Trachtenberg et al. 2002).

It remained possible that new connections were formed by single synapses and, hence, would have smaller uEPSP amplitudes and higher failure rates than pre-existing connections. However, we found that 2–4 days of whisker deprivation did not alter uEPSP amplitudes (deprived, 0.38 ± 0.11 mV, *n* = 16; control, 0.43 ± 0.11 mV, *n* = 21; *P* = 0.668, one-way ANOVA; Supplementary Table 1) (Fig. 4*A*,*B*) or EPSP failure rates (probability of failure—median [IQR]: control, 0.05 [0.02–0.79], *n* = 19 connections; 3-day trim, 0.23 [0.00–0.49], *n* = 16 connections; *P* = 0.517, Kolmogorov–Smirnov test) (Fig. 4*C*). In mature cortex, Pyr → Pyr connections in L2/3 show a mixture of facilitation and depression (Fig. 4*D* and Supplementary Fig. 5) (Cheetham et al. 2007). Comparison of the effects of whisker deprivation on the short-term synaptic dynamics is facilitated by normalization of the uEPSP amplitudes with respect to the first response in the train (uEPSP1) (Finnerty et al. 1999). We found that 2–4 days of whisker trimming did not affect the depression of the normalized steady-state amplitude when the presynaptic pyramidal neurons were stimulated to fire single action potentials at 20 Hz (Fig. 4*E*) (2- to 4-day trim, 0.87 ± 0.07, *n* = 16 connections; control, 0.88 ± 0.06, *n* = 21 connections; *P* = 0.163, one-way ANOVA). Our data show that new local excitatory connections with similar properties to control connections were formed within a few days in deprived cortex.

In contrast, after 6–8 days of whisker trimming, uEPSP amplitude was reduced (deprived, 0.11 ± 0.03 mV, *n* = 8, *P* = 0.009, one-way ANOVA) (Fig. 4*B*) and failure rates of neurotransmission were greater (probability of failure, 0.74 [0.56–0.85], *n* = 8 connections; *P* = 0.012, Kolmogorov–Smirnov test) (Fig. 4*C*). Unsurprisingly, a 20-Hz train evoked a smaller steady-state response (steady-state uEPSP amplitude, 6–8 day deprived, 0.08 ± 0.03 mV, *n* = 8; *P* = 0.001, one-way ANOVA) (Fig. 4*D*). Although the 6- to 8-day L2/3 connections in deprived cortex tended to show greater depression (Fig. 4*F*), their normalized steady-state was not statistically different from controls (6- to 8-day trim, 0.67 ± 0.08, *n* = 8 connections; control, 0.88 ± 0.06, *n* = 21 connections; *P* = 0.163, one-way ANOVA). The passive membrane properties (Supplementary Table 2) and excitability (Supplementary Fig. 6) of L2/3 pyramidal neurons in deprived cortex did not change over the experimental period. Taken together, our data show that whisker deprivation induces bidirectional changes in local excitatory connectivity in deprived cortex: Rapid formation of new local excitatory connections with similar properties to control connections is followed by weakening and loss of local excitatory connections (Fig. 4*G*). Local excitatory connections between L2/3 pyramidal neurons are typically formed by multiple synapses (Cheetham et al. 2014). The findings of reduced uEPSP amplitude and increased failure rate combined with the synaptic dynamics after 6–8 days of trimming suggest that there is loss of weaker synapses at the remaining L2/3 Pyr → Pyr connections in deprived cortex. Hence, our data suggest that the changes in local excitatory circuitry do not solely affect a subset of L2/3 Pyr → Pyr connections.

### Inhibitory Circuitry

**Figure 5.**
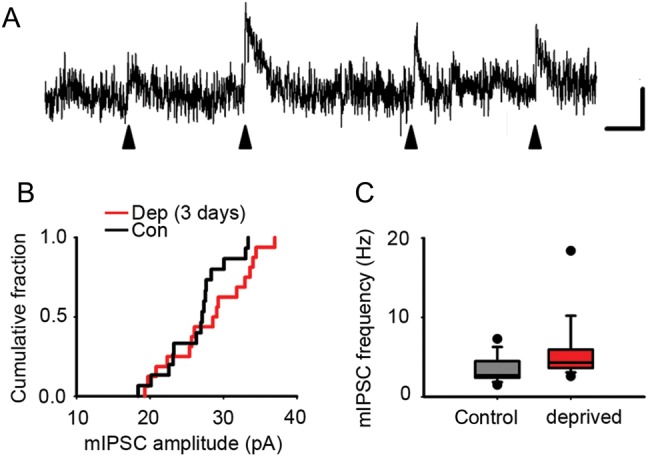
Inhibitory drive onto L2/3 pyramidal cells is not decreased by 3-day whisker deprivation. (*A*) Example trace of mIPSCs (filled arrow heads) in an L2/3 pyramidal neuron. Scale bar: 10 pA, 20 ms. (*B*) Cumulative fraction of mean mIPSC amplitude recorded from L2/3 pyramidal neurons in deprived (red) and control (black) cortex (grand mean rather than mIPSC amplitudes: deprived, 28.1 ± 1.4 pA, *n* = 16 neurons; control, 26.3 ± 1.1 pA, *n* = 15 neurons). (*C*) mIPSC frequency recorded from L2/3 pyramidal neurons in deprived (red) and control (black) cortex (mean of mean mIPSC frequencies: deprived, 4.3 [3.6–5.9] Hz, *n* = 16 neurons; control, 2.7 [2.4–4.5] Hz, *n* = 15 neurons).

It is widely thought that cortical reorganization involves changes not only in excitatory circuitry, but also in inhibitory circuitry (Jacobs and Donoghue 1991; Jones 1993; Froemke et al. 2007; Chen et al. 2011; Vogels et al. 2011; van Versendaal et al. 2012). One hypothesis is that disinhibition unmasks latent intracortical connections (Jacobs and Donoghue 1991). Therefore, we explored whether there were functional changes in inhibition in L2/3 of deprived cortex where we had found evidence of rewiring of local excitatory circuits. We first investigated whether there was a global reduction in inhibitory drive onto excitatory neurons by measuring the frequency and amplitude of mIPSCs in L2/3 pyramidal neurons. The mean mIPSC amplitude was not affected by deprivation (deprived, 28.1 ± 1.4 pA, *n* = 16 neurons; control, 26.3 ± 1.1 pA, *n* = 15 neurons; *P* = 0.33, *t*-test) (Fig. 5*A*,*B*). However, the frequency of mIPSCs was increased in deprived cortex (deprived, 4.3 [3.6–5.9] Hz, *n* = 16 neurons; control, 2.7 [2.4–4.5] Hz, *n* = 15 neurons; *P* = 0.023, Mann–Whitney rank sum test) (Fig. 5*C*). Hence, our data do not show a reduction in global inhibitory drive onto L2/3 pyramidal neurons in deprived cortex after 2–3 days of whisker trimming.

**Figure 6.**
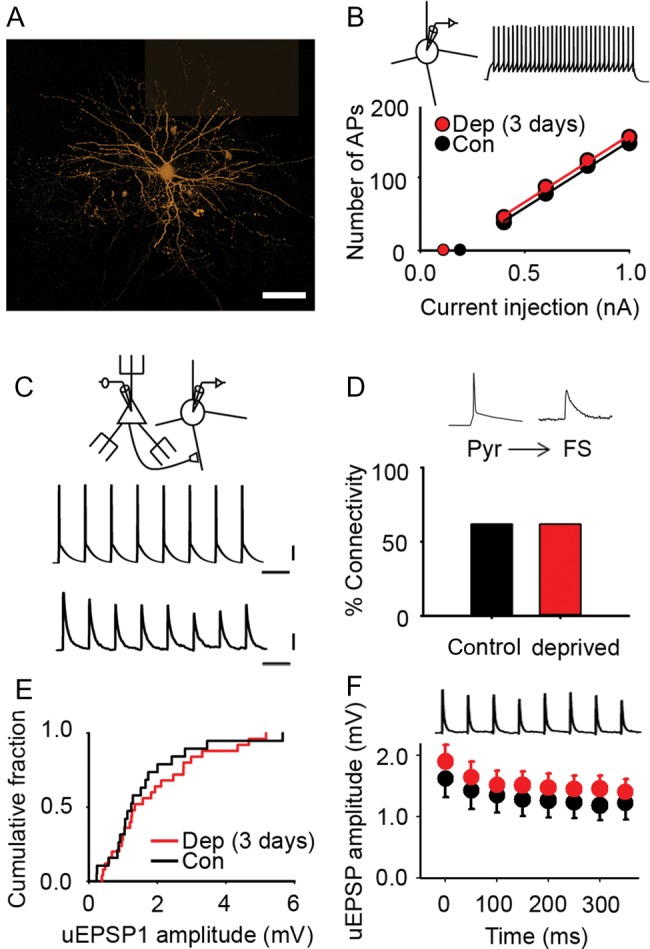
Excitatory transmission onto L2/3 FS interneurons in deprived cortex is not affected by brief sensory deprivation. (*A*) Montage of maximum intensity projections from confocal *z*-stacks of an L2/3 FS interneuron filled with AF568 (orange). Scale bar, 40 μm. (*B*) Mean number of action potentials recorded in L2/3 FS interneurons evoked by 500 ms depolarizing current pulses in control (black) and deprived (red) cortex after 2–3 days of sensory deprivation. Inset: example trace of action potentials in an L2/3 FS interneuron evoked by a +0.4-nA current pulse (500 ms). Slope of the input–output curve: deprived, 182 ± 9 action potential nA^−1^, *n* = 26 FS interneurons; control, 186 ± 10 AP nA^−1^, *n* = 32 FS interneurons. Rheobase: deprived, 0.11 ± 0.04 nA, *n* = 26 FS interneurons; control, 0.19 ± 0.03 nA, *n* = 32 FS interneurons. (*C*) Pyr → FS connection: 20 Hz train of action potentials in the presynaptic pyramidal neuron evokes short latency uEPSPs (average 50 trials) in the postsynaptic FS interneuron. Scale bars: 20 mV (top), 0.5 mV (bottom), 50 ms. (*D*) Percentage of pyramidal cell to FS interneuron pairs (Pyr → FS) that were synaptically connected in control (black, 62%) and deprived (red, 62%) cortex. (*E*) Empirical distribution plots of mean uEPSP1 amplitudes in deprived (red) and control (black) cortex. Median [IQR] of mean uEPSP1 amplitudes for Pyr → FS connections: deprived, 1.34 [0.93–2.76] mV, *n* = 25; control, 1.24 [0.88–2.00] mV; *n* = 19. (*F*) Mean uEPSP amplitude during 20 Hz trains in deprived (red, *n* = 25 Pyr → FS connections) and control (black, *n* = 19 Pyr → FS connections) cortex. Error bars, SEM.

Although global inhibitory drive was not reduced, it remained possible that whisker deprivation induced disinhibition of a subset of inhibitory circuits. It has been proposed that inhibition undergoes plastic changes to maintain the balance between excitation and inhibition in reorganizing sensory cortex (Froemke et al. 2007; Marik et al. 2010; House et al. 2011; Vogels et al. 2011). Increasing local excitatory connectivity boosts positive feedback in cortical microcircuits and amplifies signals (Douglas et al. 1995). Recurrent excitatory circuits are usually counterbalanced by feedback inhibition to maintain network stability (Shu et al. 2003). Therefore, we reasoned that increased local excitatory connectivity may be matched by parallel changes in inhibition to maintain the excitatory–inhibitory balance. We recorded from FS interneurons (Fig. 6*A*; Methods) because they have been implicated in adult cortical plasticity (Pizzorusso et al. 2002; Ruediger et al. 2011; Campanac et al. 2013). It has been proposed that the excitatory–inhibitory balance can be maintained in the hippocampus by increased excitability of FS interneurons (Campanac et al. 2013). An increase in excitability would tend to boost inhibition, whereas decreased excitability would lead to disinhibition. Whisker deprivation alters the excitability of FS interneurons in L4 of deprived cortex during development (Sun 2009). Therefore, we tested whether whisker trimming for a few days altered the excitability of FS interneurons in L2/3 of deprived cortex. We found that there was no change in the slope of the input–output curve (deprived, 182 ± 9 AP nA^−1^, *n* = 26 FS interneurons; control, 186 ± 10 action potential (PA) nA^−1^, *n* = 32 FS interneurons; *t* = 0.291, *P* = 0.77, *t*-test), rheobase (deprived, 0.11 ± 0.04 nA, *n* = 26 FS interneurons; control, 0.19 ± 0.03 nA, *n* = 32 FS interneurons; *t* = 1.556, *P* = 0.125, *t*-test), or passive membrane properties of L2/3 FS interneurons (Fig. 6*B* and Supplementary Table 3). We concluded that the excitability of L2/3 FS interneurons was not affected by a few days of whisker deprivation.

We next made electrophysiological recordings from synaptically connected pairs of neurons comprising an L2/3 FS interneuron and an L2/3 pyramidal neuron to investigate whether whisker deprivation altered local inhibitory circuits. We first considered the excitation of FS interneurons. Our recordings had shown a marked increase in Pyr → Pyr connectivity in L2/3 of deprived cortex after 3 days of whisker deprivation. In contrast, we found that Pyr → FS connectivity in deprived cortex was unchanged (Pyr → FS connectivity: 3-day deprived, 62%, 26/42 pairs tested; control, 62%, 26/42 pairs tested; *P* = 1.00, *χ*^2^ test) (Fig. 6*C*,*D*). We concluded that the elevated Pyr → Pyr connectivity following 3 days of whisker deprivation was not part of a generalized, nonspecific increase in local excitatory connectivity.

Although Pyr → FS connectivity had not changed, it remained possible that existing Pyr → FS connections were modified following whisker trimming. Therefore, we compared the amplitudes of the first uEPSP (uEPSP1) in an L2/3 FS interneuron evoked by a train of action potentials in a presynaptic L2/3 pyramidal neuron (Fig. 6*C*). We found that 3 days of whisker trimming did not alter the amplitude of the uEPSP1 in L2/3 FS interneurons in deprived cortex (deprived, 1.34 [0.93–2.76] mV, *n* = 25 Pyr → FS connections; control, 1.24 [0.88–2.00] mV; *n* = 19 Pyr → FS connections; *P* = 0.51, Mann–Whitney rank sum test) (Fig. 6*E*). Similarly, the synaptic dynamics of Pyr → FS connections during a 20-Hz train were unchanged (Fig. 6*F* and Supplementary Fig. 7*A*) (normalized steady-state amplitude: deprived: 0.74 ± 0.04; *n* = 25; control: 0.76 ± 0.05, *n* = 19; *P* = 0.70, *t*-test). Hence, our data suggested that 3 days of whisker deprivation was not accompanied by increased excitation of L2/3 FS interneurons by neighboring pyramidal neurons.

**Figure 7.**
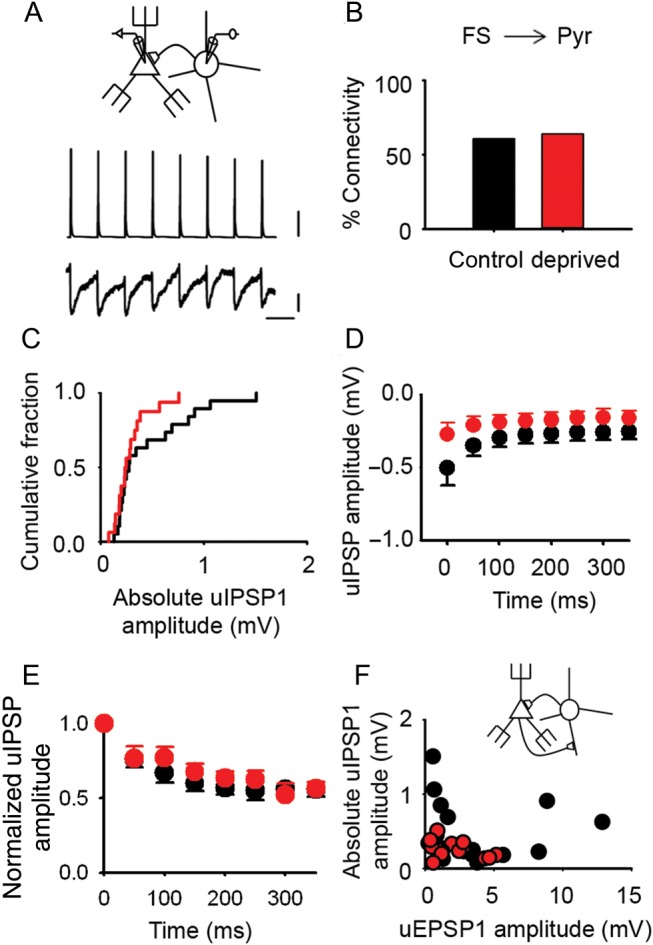
Inhibitory transmission onto pyramidal neurons in L2/3 of deprived cortex is unaltered by short periods of whisker deprivation. (*A*) Schematic showing an FS interneuron synaptically connected to a pyramidal cell (top); train of 8 action potentials in the presynaptic FS interneuron generates 8 short latency uIPSPs in the postsynaptic pyramidal neuron (average of 50 trials). Scale bars (top to bottom): 20 mV, 0.1 mV, 100 ms. (*B*) Percentage of tested FS interneuron to pyramidal cell pairs (FS → Pyr) that were synaptically connected in control (black, 62%) and deprived (red, 64%) cortex. (*C*) Empirical distribution plots of the amplitudes of mean uIPSP1 (absolute value) in deprived (red) and control (black) cortex. Median [IQR] of mean uIPSP1 amplitudes: deprived: −0.24 [−0.35 to −0.18] mV, *n* = 16 FS → Pyr connections; control, −0.27 [−0.69 to −0.20] mV, *n* = 19 FS → Pyr connections. (*D*) Mean uIPSP amplitude during 20 Hz trains in deprived (red, *n* = 8 FS → Pyr connections) and control (black, *n* = 13 FS → Pyr connections) cortex. Error bars, SEM. (*E*) uIPSP amplitudes during a 20-Hz train normalized to uIPSP1 for each L2/3 FS → Pyr connection in 3-day deprived cortex (red, *n* = 8) and in control cortex (black, *n* = 13). Error bars are within the majority of circles. (*F*) Relationship between mean uEPSP1 amplitude and mean uIPSP1 amplitude (absolute values) for pairs of reciprocally connected FS interneurons and pyramidal cells in control (black) and deprived (red) cortex (correlation: deprived, *r* = −0.40, *n* = 14 reciprocally connected FS → Pyr pairs; control, *r* = −0.06, *n* = 17 reciprocally connected FS → Pyr pairs).

We next considered inhibition of pyramidal neurons by FS interneurons (FS → Pyr) since strengthening of inhibitory synapses has been predicted to play a role in maintaining the excitatory–inhibitory balance during cortical reorganization (Vogels et al. 2011). uIPSPs were measured by holding the L2/3 pyramidal neuron at −55 mV in current clamp mode while stimulating the presynaptic FS interneuron to fire a train of action potentials (Fig. 7*A*). The probability of finding an FS → Pyr connection was similar in control and in deprived cortex (deprived: 64%, 16/25 connections tested; control, 62%, 17/28 connections tested; *P* = 0.97, *χ*^2^ test) (Fig. 7*B*). The mean uIPSP amplitude in deprived L2/3 pyramidal neurons after a 3-day deprivation was not different from controls (deprived: −0.24 [−0.35 to −0.18] mV; *n* = 16 connections; control, −0.27 [−0.69 to −0.20] mV; *n* = 19 connections; *P* = 0.13, *t*-test after log transformation of the absolute uIPSP amplitude) (Fig. 7*C*). The synaptic dynamics during a 10-Hz train showed minimal and variable depression of uIPSPs during the train (Supplementary Fig. 7*B*,*C*). uIPSPs evoked by 20 Hz trains showed greater synaptic depression than the responses elicited by 10 Hz stimulation (Fig. 7*D* and Supplementary Fig. 7). However, 3-day whisker deprivation did not affect the short-term synaptic dynamics of uIPSPs evoked by 20 Hz trains (Fig. 7*E*) (normalized steady-state amplitude: deprived, 0.57 ± 0.05, *n* = 8 FS → Pyr connections; control, 0.56 ± 0.05, *n* = 13 FS → Pyr connections; *P* = 0.842, *t*-test). Whisker deprivation for 3 days did not change the reversal potential for uIPSPs (Supplementary Material). Taken together, our findings suggest that 3 days of whisker deprivation did not alter the strength of inhibition from L2/3 FS interneurons onto L2/3 pyramidal neurons as a whole.

Our analysis up till this point has studied feedback inhibition at a population level. It remains possible that inhibition from a subset of FS interneurons was modified. In principle, inhibitory plasticity mechanisms in mature cortex could regulate feedback inhibition at the level of each disynaptic circuit (Kullmann et al. 2012). In simple, disynaptic negative-feedback circuits, the strength of excitation of the FS interneuron by the pyramidal cell (Pyr → FS), and the strength of inhibition of the pyramidal cell by the FS interneuron (FS → Pyr) may move in parallel when feedback inhibition either increases or decreases. Altered inhibition could then manifest as a perturbation of this relationship. We investigated this idea in the subset of FS interneuron–pyramidal cell pairs that were reciprocally connected. We found that the amplitude of the mean uEPSP1 was not correlated with the mean uIPSP1 amplitude in either control or deprived cortex (control, *r* = −0.06, *n* = 17 reciprocally connected FS → Pyr pairs, *P* = 0.796, Pearson correlation; deprived, *r* = −0.40, *n* = 14 reciprocally connected FS → Pyr pairs; *P* = 0.159, Pearson Correlation) (Fig. 7*F*). We concluded that the strength of feedback inhibition was not regulated at the level of single recurrent feedback circuits in mature cortex.

## Discussion

We investigated how mature sensory cortex reorganizes over the first few days after its principal sensory input is lost. Our findings show that local excitatory circuits undergo bidirectional rewiring in somatosensory cortex that has been deprived of its principal whisker input. Here, we use rewiring to mean the formation of entirely new connections or loss of established connections rather than the formation and loss of synapses, which can occur at existing connections. Specifically, we found a rapid 3-fold increase in connectivity between L2/3 pyramidal neurons in deprived cortex. This increased connectivity was followed within days by a loss of excitatory connections, which returned local excitatory connectivity in L2/3 to baseline levels. This rewiring does not represent a nonspecific increase in connectivity as inhibitory circuits involving FS interneurons were not affected. The location and temporal profile of the changes in local excitatory connectivity were consistent with our BOLD fMRI findings. Our findings suggest that the rewiring reconfigures local excitatory circuits in deprived cortex (Fig. 4*G*).

### Experience-Dependent Rewiring of Local Excitatory Circuits

Altered sensory experience increases synapse formation and elimination in cortical circuits (Holtmaat and Svoboda 2009; Barnes and Finnerty 2010). However, the effects of synapse formation and elimination on the wiring diagram for cortical circuits remain unclear. This arises because the identities of both the presynaptic and the postsynaptic neuron are not usually known. For instance, longitudinal imaging in vivo has revealed that altered sensory experience and learning promotes rewiring through increased turnover of dendritic spines on pyramidal neurons in adult neocortex (Holtmaat and Svoboda 2009). However, the presynaptic neurons that synapse with the new spines are not usually identified. Hence, it is not clear whether new synapses are formed between neurons with an existing synaptic connection or whether the new synapse wires up neurons that were previously unconnected. The formation of excitatory connections between previously unconnected neurons has been inferred from axonal growth into new brain regions (Darian-Smith and Gilbert 1994; Jones 2000; Marik et al. 2010) and loss of connections has been deduced from retrenchment of axonal arbors (Antonini et al. 1998; Wimmer et al. 2010; Oberlaender et al. 2012). Again, however, the postsynaptic partners of the restructured axon are not usually known. Our electrophysiological recordings between identified neurons show that new excitatory connections are formed between pyramidal neurons that were previously unconnected. Formation of entirely new connections between pyramidal neurons exceeds loss of existing connections in the first few days of whisker deprivation with the result that connectivity increases local excitatory circuits in deprived cortex.

Local excitatory circuits can form new excitatory connections rapidly because of the geometry of the axons and dendrites of L2/3 neurons. In a barrel column, the axon of an L2/3 pyramidal neuron lies very close to the dendrites of a neighboring pyramidal neuron at multiple points (Cheetham et al. 2008). The proximity of axons and dendrites enables new synapses to be formed by outgrowth of a dendritic spine to contact a nearby axon (Knott et al. 2006). Accordingly, extensive growth of axons is not required. The formation of entirely new connections between L2/3 pyramidal neurons is facilitated by the increase in turnover of dendritic spines induced by whisker trimming (Trachtenberg et al. 2002). New synapses require approximately 1 day before they are functional (Nagerl et al. 2007). Hence, new multisynaptic connections between L2/3 pyramidal neurons can be formed within a few days as our recordings found.

It has been proposed that cortical maps expand through unmasking of latent intracortical connections. However, silent synapses are rare in mature neocortex (Barnes and Finnerty 2010) and we found no evidence of disinhibition. Therefore, our results suggest that unmasking of latent intracortical connections contributes little to the reorganization of L2/3 deprived cortex.

Long-lasting expansion of cortical maps has been attributed to invasion of deprived cortex by axons of L2/3 pyramidal neurons in spared cortex (Darian-Smith and Gilbert 1994; Jones 2000). Yet, longitudinal imaging indicates that this takes weeks (Marik et al. 2010). Therefore, invasion of deprived cortex by axons from spared cortex may drive the loss of local excitatory connections in deprived cortex, which continues for many weeks after our study period (Cheetham et al. 2007). However, the temporal progression of the axonal growth suggests that it is not primarily responsible for the rapid expansion of BOLD whisker maps after 3 days of trimming.

### Cortical Reorganization Imaged with BOLD fMRI

The spatial extent of the expanded BOLD whisker representations that we describe is of the order of one to a few barrel columns and is in broad agreement with other fine-scale studies of whisker map reorganization in mature animals (Diamond et al. 1994; Glazewski and Fox 1996; Polley et al. 1999). Our BOLD fMRI data did not show a monotonic expansion of the spared whisker representations, but instead revealed a rapid expansion followed by a retraction of the BOLD whisker representation. Our results are similar to the findings from studies of perceptual learning, which show map expansion during learning followed by map retraction after the task is learnt (Molina-Luna et al. 2008; Yotsumoto et al. 2008; Reed et al. 2011; Gilbert and Li 2012).

The effect of altered sensory experience on the thalamocortical input to whisker barrel cortex has been studied with several techniques including BOLD fMRI. The results vary with the experimental protocol and whether spared or deprived cortex is studied. A lesion study has reported that the BOLD signal in spared SI is enhanced 2 weeks after the lesion, and that this is associated with strengthening of the thalamocortical input to L4 whisker barrels (Yu et al. 2012). The potentiated thalamic input was attributed to an increased number and greater strength of thalamocortical synapses (Yu et al. 2012). In contrast, we found that nontraumatic whisker trimming did not affect the amplitude of the BOLD signal, although the BOLD representation of spared whiskers was expanded. Furthermore, anatomical studies show loss of thalamocortical axon branches in deprived L4 barrels after 3 days of whisker trimming (Wimmer et al. 2010; Oberlaender et al. 2012). Bouton density is unchanged (Wimmer et al. 2010; Oberlaender et al. 2012), suggesting that the number of thalamocortical synapses in deprived whisker barrels is decreased. Hence, the expanded BOLD whisker representation that we report is not attributable to increased numbers of strengthened thalamocortical inputs to L4 deprived cortex.

### Inhibition and Cortical Plasticity

It has been hypothesized that inhibition may play a role in adult cortical reorganization through a sustained period of disinhibition (Jacobs and Donoghue 1991; Chen et al. 2011; Keck et al. 2011; van Versendaal et al. 2012). However, we found no evidence for global disinhibition in deprived cortex after 3 days of whisker trimming. Instead, we found an increase in the frequency of mIPSCs and no change in mIPSC amplitude in L2/3 pyramidal neurons in deprived cortex. Our mIPSC data indicated that the number of inhibitory synapses onto L2/3 pyramidal neurons was increased or that the number of neurotransmitter release sites at inhibitory synapses was greater. We investigated whether a subset of interneurons may be affected by sensory experience, focusing on FS interneurons because they have been implicated in adult cortical reorganization (Pizzorusso et al. 2002; Ruediger et al. 2011; Campanac et al. 2013). However, feedback inhibition involving FS interneurons was normal. Our results were surprising since the rapid structural changes to inhibitory circuits in deprived cortex reported in other studies would appear to indicate disinhibition with loss of inhibitory boutons (Marik et al. 2010; Keck et al. 2011), remodeling of the axonal arbors, and retraction of the dendritic tips of L2/3 interneurons (Marik et al. 2010; Chen et al. 2011). Collectively, the structural plasticity of L2/3 interneurons and our data suggest that there is a redistribution of inhibitory synapses across the dendritic tree of L2/3 pyramidal neurons in deprived cortex rather than an absolute loss of inhibitory input. The increase in mIPSC frequency could arise if the formation of new inhibitory synapses exceeded the elimination of existing inhibitory synapses. Finally, the redistribution of inhibitory synapses may be coordinated with the reorganization of local excitatory circuits (Chen et al. 2012).

Our findings cannot exclude a role for disinhibition in deprived cortex during cortical reorganization. We focused on global inhibitory drive in L2/3 pyramidal neurons (mIPSC data) and plasticity within local inhibitory circuits involving FS interneurons. It remains possible that another group of interneurons that we did not record from, for example, somatostatin-positive interneurons, are selectively disinhibited during cortical map plasticity. An alternative hypothesis is that disinhibition is transient to enable strengthening or remodeling of excitatory circuitry (Froemke et al. 2007; Letzkus et al. 2011; Vogels et al. 2011). Strengthening of excitatory synapses is followed within hours by strengthening of inhibitory circuitry to maintain the balance between excitation and inhibition in auditory cortex (Froemke et al. 2007; Vogels et al. 2011). The effect of increasing recurrent excitation in L2/3 on the excitatory–inhibitory balance in deprived cortex is uncertain. Our data suggest that excitation and inhibition are not balanced at the level of disynaptic, feedback inhibitory circuits. Recurrent excitation is a form of positive feedback circuit (Douglas et al. 1995) and would, therefore, tend to increase firing of pyramidal neurons. However, this effect may be offset by the reduction in neural activity in deprived cortex following the loss of its principal sensory input. Therefore, information processing may not require adjustments to inhibitory circuitry.

### Rewiring Mature Cortical Microcircuits

Why do local excitatory circuits rewire in L2/3 of deprived cortex? In mature animals, the rewiring modifies established local excitatory circuits. Pyramidal neurons in these circuits are not connected randomly with neighboring pyramidal neurons (Song et al. 2005; Yoshimura et al. 2005; Cheetham et al. 2007), and the probability of finding a connection between neighboring L2/3 pyramidal neurons in SI is relatively low despite the proximity of the axon and dendrites of neighboring L2/3 pyramidal neurons (Cheetham et al. 2008). These features suggest that the configuration of the wiring in mature local excitatory circuits is important for the function of those circuits.

In vivo calcium imaging of L2/3 pyramidal neurons during whisker deprivation indicates that previously inactive, “silent” neurons are recruited into local excitatory networks during cortical plasticity (Margolis et al. 2012). If a pyramidal neuron was already part of a network, then adjusting the strength of existing connections in the network could boost the firing of that neuron. However, this strategy may not be an effective way to recruit “silent” neurons to a cortical microcircuit when excitatory connectivity is low, as occurs in L2/3 of somatosensory cortex (Cheetham et al. 2008). The increase in local excitatory connectivity that we describe offers a mechanism whereby silent neurons can be recruited into cortical microcircuits. The subsequent loss of excitatory connections enables those local excitatory circuits to be refined (Fig. 4*G*). We propose that the reconfiguration of local excitatory circuits facilitates the redistribution of neural firing during cortical plasticity.

## Supplementary Material

Supplementary material can be found at: http://www.cercor.oxfordjournals.org/.

## Funding

The work was funded by the Wellcome Trust (B.d.C.A., GR068636MA and Senior Clinical Fellowship awarded to G.T.F., 061135/Z) and MRC (G.A., S.J.B., and B.d.C.A.). Funding to pay the Open Access publication charges for this article was provided by the Wellcome Trust.

## Supplementary Material

Supplementary Data
