# Supplementary material for: Rapid Bidirectional Reorganization of Cortical Microcircuits
Source: Cereb Cortex. 2014 May 16;25(9):3025–35. doi: 10.1093/cercor/bhu098 (PMC4537443; doi:10.1093/cercor/bhu098)
Supplement: Supplementary Data [file supp_25_9_3025__index.html]

Rapid Bidirectional Reorganization of Cortical Microcircuits — Supplementary Data 

# Rapid Bidirectional Reorganization of Cortical Microcircuits

## Supplementary Data

Supplementary Data

**Files in this Data Supplement:**

- Supplementary Data - Pdf file
