# Supplementary material for: Rapid Bidirectional Reorganization of Cortical Microcircuits
Source: Cereb Cortex. 2014 May 16;25(9):3025–35. doi: 10.1093/cercor/bhu098 (PMC4537443; doi:10.1093/cercor/bhu098)
Supplement: Supplementary Data [file supp_bhu098_bhu098supp.pdf]

SUPPLEMENTARY MATERIAL

SUPPLEMENTARY DATA

SI positive BOLD response (PBR)

The peak PBR was in upper cortical layers, but rarely within 100 μm of the pia (4 of 23 in SI of control animals) (Supplementary Fig. 2), suggesting that the positive BOLD signal was generated within the cortex.

The location of the peak positive BOLD response and the centre of mass of the BOLD response were located at the same site. In whisker trimmed animals, there was a tendency for both indices to be displaced along the cortical surface into the deprived D – E barrel columns. This was not statistically significant after 3 days of trimming, but became so after 7 days of whisker trimming (1 mm displacement; $P < 0.05$, Kruskal-Wallis ANOVA on ranks). This result should be interpreted cautiously because: there is significant variability in the location of the peak PBR; the whisker barrel column rows run in and out of the image plane, but are not perpendicular to it; and enhanced activity in the caudal C-row whiskers could cause the peak BOLD response to shift medially.

Synaptically-connected L2/3 pyramidal neurons

Connectivity between control neurons and uEPSP amplitude did not change between the P32 – P34 and P36 – 38 groups ($P = 0.686$, Chi-square) consistent with previous work (Cheetham et al., 2007; Cheetham et al., 2008). Therefore, control recordings were pooled. Neither whisker trimming nor age affected the passive membrane properties of L2/3 pyramidal neurons (Supplementary Table 1).

Dendritic spine density

We counted dendritic spines on L2/3 pyramidal neurons to give a structural measure of synapse number (Methods). There was no change in spine density along the basal dendrites (DEP coefficient, 0.007 ± 0.022 spines.μm^{-1}, P = 0.749, generalized additive model; n = 64 basal dendrites from eight deprived L2/3 pyramidal neurons and 53 basal dendrites from seven control L2/3 pyramidal neurons) or along the apical main stem (DEP coefficient, 0.017 ± 0.041 spines.μm^{-1}, P = 0.686, generalized additive model; n = 62 apical dendrite branches from seven deprived L2/3 pyramidal neurons and 49 apical dendrite branches from five control L2/3 pyramidal neurons) (Supplementary Fig. 3). We concluded that spine density was not affected by 2 – 4 days of whisker trimming.

mEPSPs after 2 – 4 days of whisker trimming

The mean amplitude of mEPSPs in deprived L2/3 pyramidal neurons after 2 – 4 days of trimming was not different from control values (deprived, 0.17 ± 0.01 mV, n = 20; control, 0.15 ± 0.01 mV, n = 11; P = 0.281, t-test) (Supplementary Fig. 4A, B). Similarly, the mean frequency of mEPSPs per neuron was not affected by 2 – 4 days of whisker trimming (deprived, 15.0 ± 1.5 Hz, n = 20; control, 11.1 ± 1.2 Hz, n = 11: P = 0.088, t-test) (Supplementary Fig. 4C).

Steady state uEPSP amplitude at Pyr → Pyr connections

The steady-state amplitude of uEPSPs evoked at L2/3 Pyr → Pyr connections during a 20 Hz train was similar in control cortex and after 2 – 4 days deprivation (Fig. 4D) (control, 0.34 ± 0.07 mV, n = 21; 2 – 4 day trim, 0.30 ± 0.08 mV, n = 16; P = 0.647, one-way ANOVA).

uIPSP reversal potential

The uIPSP reversal potential was calculated from FS – Pyr pairs in control and deprived cortex. The membrane potential of pyramidal cells was varied in 5 to 10 mV steps from -50 mV to -90 mV. The amplitude of the uIPSP was recorded at each holding potential. A quadratic function ($f(x) = c + ax + bx^2$) was fitted to the data. The reversal potential for each connection was calculated from the fit as the holding potential that resulted in a uIPSP amplitude of 0 mV. We found that the mean reversal potential for uIPSP in deprived connections was not different from control values (deprived, -66.0 ± 0.9 mV, n = 4 FS → Pyr connections; control, -66.5 ± 1.1 mV, n = 5 FS → Pyr connections; P = 0.77, t- test).

Supplementary Table 1 Passive membrane properties of pairs of synaptically-connected L2/3 pyramidal neurons

	2 – 4 day trim		6 – 8 day trim	
	Control (n = 11)	Deprived (n = 16)	Control (n = 10)	Deprived (n = 8)
Presynaptic resting membrane potential (mV)	-72 ± 2	-73 ± 2	-69 ± 3	-73 ± 3
Presynaptic input resistance (MΩ)	64 ± 5	54 ± 3	54 ± 6	65 ± 7
Presynaptic membrane time constant (ms)	15.1 ± 1.1	13.5 ± 0.8	14.8 ± 1.4	13.2 ± 1.3
Presynaptic membrane capacitance (pF)	250 ± 20	260 ± 20	280 ± 30	220 ± 30
Postsynaptic resting membrane potential (mV)	-76 ± 2	-73 ± 1	-73 ± 2	-76 ± 2
Postsynaptic input resistance (MΩ)	63 ± 5	54 ± 4	56 ± 5	74 ± 6
Postsynaptic membrane time constant (ms)	15.0 ± 1.1	12.5 ± 0.9	13.7 ± 1.1	14.9 ± 1.5
Postsynaptic membrane capacitance (pF)	240 ± 20	250 ± 20	250 ± 20	210 ± 30

Deprivation did not affect (two-way ANOVA): presynaptic input resistance (P = 0.869, $F_{(1,41)}$ = 0.027), presynaptic membrane time constant (P = 0.189, $F_{(1,41)}$ = 1.789), postsynaptic resting membrane potential (P = 0.967, $F_{(1,41)}$ = 0.002), postsynaptic membrane time constant (P = 0.538, $F_{(1,41)}$ = 0.385), postsynaptic membrane capacitance (P = 0.508, $F_{(1,41)}$ = 0.446), presynaptic membrane capacitance (P = 0.333, $F_{(1,41)}$ = 0.960), presynaptic resting membrane

potential (P = 0.277, $F_{(1,41)}$ = 1.216) or postsynaptic input resistance (P = 0.791, $F_{(1,41)}$ = 0.071).

Age did not affect (two-way ANOVA): presynaptic input resistance (P = 0.966, $F_{(1,41)}$ = 0.002), presynaptic membrane time constant (P = 0.804, $F_{(1,41)}$ = 0.062), postsynaptic resting membrane potential (P = 0.795, $F_{(1,41)}$ = 0.069), postsynaptic membrane time constant (P = 0.631, $F_{(1,41)}$ = 0.234), postsynaptic membrane capacitance (P = 0.599, $F_{(1,41)}$ = 0.280), presynaptic membrane capacitance (P = 0.976, $F_{(1,41)}$ = 0.001), presynaptic resting membrane potential (P = 0.451, $F_{(1,41)}$ = 0.579), postsynaptic input resistance (P = 0.315, $F_{(1,41)}$ = 1.035).

Supplementary Table 2 Passive membrane properties of L2/3 pyramidal neurons used for excitability analysis

	2 – 4 day trim		6 – 8 day trim	
	Control<br>(n = 32)	Deprived<br>(n = 34)	Control<br>(n = 21)	Deprived<br>(n = 26)
Resting membrane potential (mV)	-75<br>[-79 – -70]	-76<br>[-80 – -73]	-74<br>[-77 – -73]	-75<br>[-78 – -70]
Input resistance (MΩ)	52 ± 3	55 ± 3	54 ± 4	58 ± 3
Membrane time constant (ms)	13.6 ± 0.7	13.2 ± 0.6	12.7 ± 0.8	13.7 ± 0.7
Membrane capacitance (pF)	250<br>[220 – 300]	250<br>[190 – 320]	240<br>[190 – 290]	220<br>[200 – 290]

Deprivation status did not affect: input resistance (P= 0.244, $F_{(1,39)}$ = 1.371, two-way ANOVA), membrane time constant (P = 0.649, $F_{(1,39)}$ = 0.209, two-way ANOVA), resting membrane potential (P = 0.509 after 2 – 4 days of whisker trimming, P = 0.932 after 6 – 8 days of whisker trimming, MWRST) and membrane capacitance (P = 0.397 after 2 – 4 day trim, P = 0.991 after 6 – 8 day trim, MWRST).

Supplementary Table 3 Passive membrane properties of L2/3 FS interneurons used for excitability analysis

FS interneurons	Control (n = 30)	Deprived (n = 38)	Statistics
Resting membrane potential (mV)	-65 ± 1	-67 ± 1	p = 0.268 (t-test**)**
Input resistance (MΩ)	58 [46 – 73]	51 [44 – 62]	p = 0.224 (Mann-Whitney**)**
Membrane time constant (ms)	5.3 ± 0.2	4.9 ± 0.2	p = 0.268 (t-test**)**
Membrane capacitance (pF)	92 [69 – 104]	85 [75 – 104]	p = 0.607 (Mann-Whitney**)**

Deprivation did not affect the resting membrane potential (Vm), input resistance (Rm), membrane capacitance (Cm) and time constant (τm) of L2/3 FS interneurons compared with controls.

SUPPLEMENTARY FIGURES

Supplementary Figure 1 Axo-axonic cell

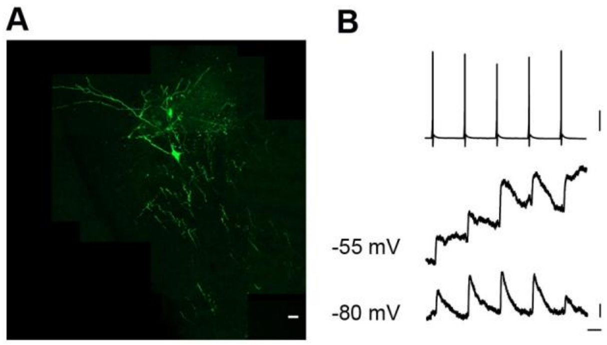

(**A**) Montage of maximum intensity projections from confocal *z*-stacks of a L2/3 axo-axonic cell (chandelier cell) filled with AF488 (green). The pia is towards the top left of the panel. Scale bar, 20 μm. (**B**) Train of 5 action potentials in the presynaptic axo-axonic cell generates 5 short-latency depolarixing uIPSPs in the postsynaptic L2/3 pyramidal neuron at resting membrane potential (-80 mV). When the membrane potential is held at -55 mV, uIPSPs are still positive going, depolarising the postsynaptic pyramidal neuron towards firing threshold. Scale bars, top to bottom: 20 mV, 0.1 mV, 20 ms.

Supplementary Figure 2 Location of peak BOLD signal following whisker trimming

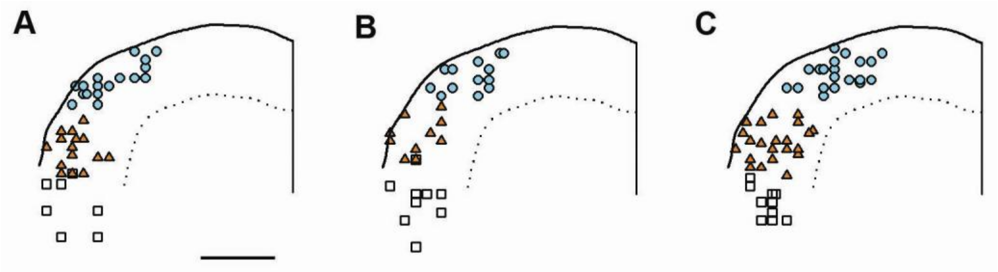

(**A**) Projections of the locations of the peak PBRs in SI (cyan circles), SII (orange triangles) and parietal ventral area (open squares) after sham-trimming (n = 23 rats) onto a coronal image slice through whisker barrel cortex. Calibration bar, 2 mm. The midline is denoted by the vertical line. The pia is shown as a solid line and the junction between neocortex and white matter by a dashed line. (**B**) Projections of the locations of the peak PBRs in SI (cyan circles), SII (orange triangles) and parietal ventral area (open squares) after a 3-day trim (n = 14 rats) onto a coronal image slice through whisker barrel cortex. (**C**) Projections of the locations of the peak PBRs in SI (cyan circles), SII (orange triangles) and parietal ventral area (open squares) after a 7-day trim (n = 28 rats) onto a coronal image slice through whisker barrel cortex. Not all points are visible due to overlap.

Supplementary Figure 3 Dendritic spine density on L2/3 pyramidal neurons does not increase in deprived cortex

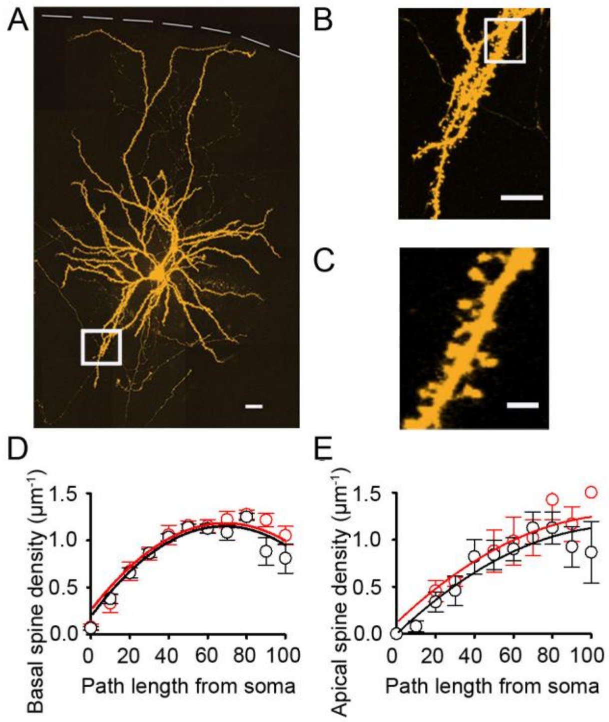

(**A**) Maximum intensity projection of a L2/3 pyramidal neuron. Scale bar, 15 µm. (**B**) Enlargement of basal dendrite in white box in A. Scale bar, 10 µm. (**C**) Single confocal section through dendrite in white box in B. Scale bar, 2 µm. (**D**) Mean spine density along basal dendrites after 2 – 4 days of whisker trimming (red) and controls (open circles). Spine counts: n = 64 basal dendrites from eight deprived L2/3 pyramidal neurons and 53 basal dendrites from seven control L2/3 pyramidal neurons. (**E**) Mean spine density along apical main stem after 2 – 4 days of whisker trimming (red) and controls (open circles). Spine counts: n = 62 apical dendrite branches from seven deprived L2/3 pyramidal neurons and 49 apical dendrite branches from five control L2/3 pyramidal neurons. The fits from the statistical model to deprived (red) and control (black) spine densities are overlaid in (D) and (E).

Supplementary Figure 4 mEPSP amplitude and frequency in deprived L2/3 pyramidal neurons are not altered after 2 – 4 days of whisker trimming.

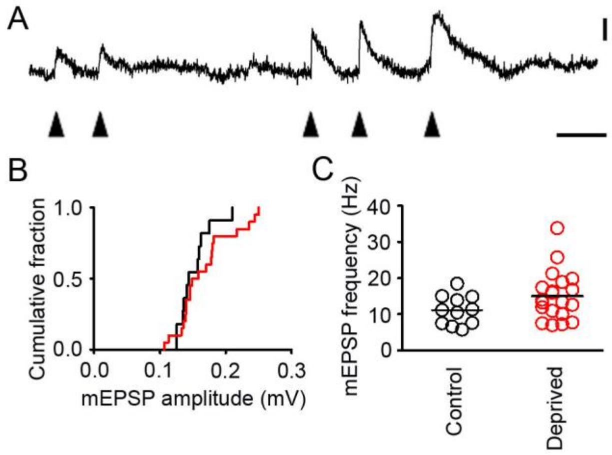

(**A**) Example trace of mEPSPs (filled arrows). Filled arrowheads denote mEPSP onset. Scale bars: 0.2 mV, 50 ms. (**B**) Cumulative fraction of mean mEPSP amplitude per neuron after 2 – 4 days of whisker trimming (red) and for age-matched control values (black) (mean of mean mEPSP amplitudes: 2 – 4 day trim, 0.17 ± 0.01 mV, n = 20 neurons; control, 0.15 ± 0.01 mV, n = 11 neurons). (**C**) Mean frequency of mEPSPs in neurons deprived for 2 – 4 days (red circles) and in control neurons (unfilled circles). Horizontal lines denote the group means (2 – 4 day trim, 15.0 ± 1.5 Hz, n = 20 neurons; control, 11.1 ± 1.2 Hz, n = 11 neurons).

Supplementary Figure 5 Pyr → Pyr connections showing synaptic depression in L2/3 of deprived cortex

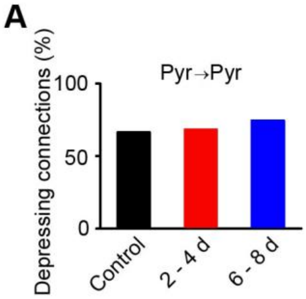

(**A**) Percentage of L2/3 Pyr → Pyr connections in deprived cortex showing synaptic depression after 2 – 4 days of trimming (61 %, χ^2 = 0.021, df = 1,P = 0.886) or after 6 – 8 days of trimming (75 %, χ^2 = 0.020, df = 1,P = 0.890) were similar to control L2/3 Pyr → Pyr connections (67%).

Supplementary Figure 6 Excitability of L2/3 pyramidal neurons in deprived cortex

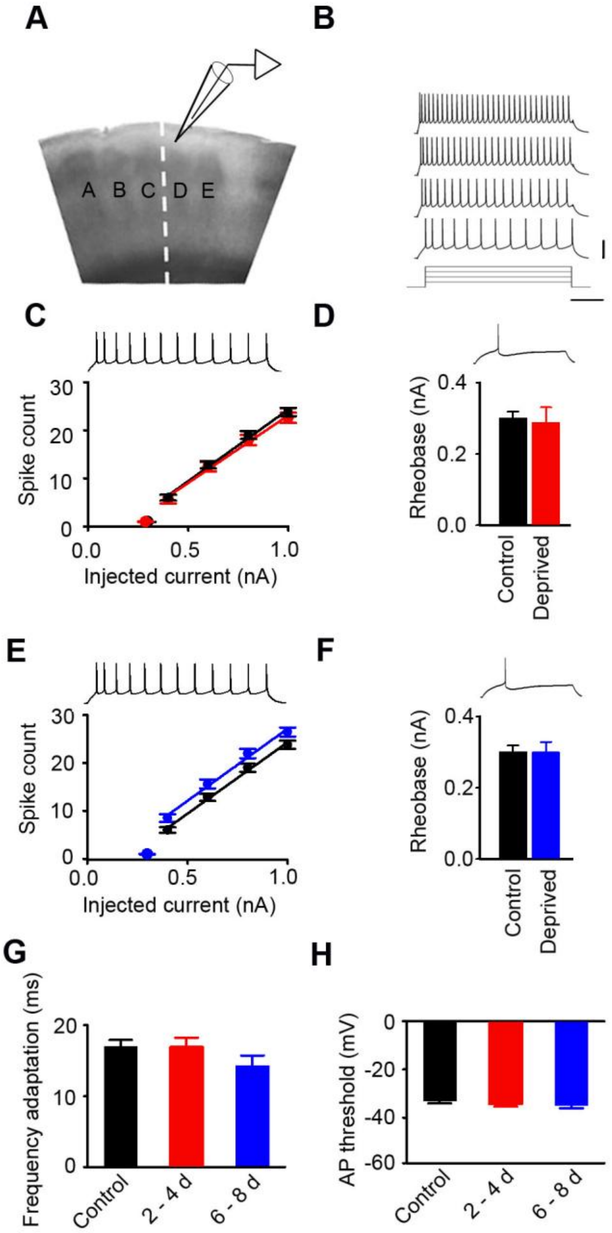

(**A**) Diagram depicting recording of single L2/3 pyramidal neurons in deprived cortex. White dashed line denotes the boundary between spared and deprived cortex. (**B**) Action potentials evoked in a L2/3 pyramidal neuron by 500 ms current pulses (+0.4 to +1.0 nA). Scale bars: 50 mV, 100 ms. (**C**) Input – output curve for firing of L2/3 pyramidal neurons after 2 – 4 days deprivation and in age-matched control cortex. The slope of the line was not altered by 2 – 4 days of whisker trimming (deprived, 56 ± 2 action potentials.$nA^{-1}.s^{-1}$, n = 24 neurons;

control, 60 ± 2 action potentials.$nA^{-1}.s^{-1}$, n = 41 neurons; P = 0.162, t-test). (**D**) Rheobase was not changed after 2 – 4 days deprivation (rheobase: deprived, 0.29 ± 0.04 nA, n = 10 neurons; control, 0.30 ± 0.02 nA, n = 26 neurons, P= 0.768, t-test). (**E**) Input – output curve for firing of L2/3 pyramidal neurons after 6 – 8 days deprivation and in age-matched control cortex. The slope of the input – output line was unchanged (deprived, 57 [55 – 63] action potentials.$nA^{-1}.s^{-1}$, n = 33 neurons; control, 60 [54 – 66] action potentials.$nA^{-1}.s^{-1}$, n = 41 neurons; P = 0.510, Mann-Whitney Rank Sum test). (**F**) Rheobase was not changed after 6 – 8 days of whisker trimming (rheobase: deprived 0.30 ± 0.03 nA, n= 16 neurons; control 0.30 ± 0.02 nA, n = 26 neurons, P = 0.980, t-test). (**G**) Adaptation of action potential firing was similar in L2/3 pyramidal neurons across the experimental time period (adaptation calculated as steady-state interspike interval minus initial interspike interval during a + 1.0 nA, 500 ms current pulse: control, 15 [13 – 19] ms, n = 41 neurons; 2 - 4 days deprivation, 16 [12 – 19] ms, n = 24 neurons; 6 – 8 days deprivation, 15 [12 – 17] ms, n = 33 neurons, P = 0.524 ANOVA on Ranks). (**H**) Action potential threshold did not change across the experimental time period (threshold: control, -35 [-38 – -28] mV, n = 41 neurons, 2 – 4 days deprivation, -35 [-39 – -32] mV, n = 24 neurons; 6 – 8 days deprivation, -35 [-39 – -32] mV, n= 33 neurons, P = 0.589, ANOVA on Ranks).

Supplementary Figure 7 Normalized synaptic dynamics of Pyr → FS and of FS → Pyr connections in 3-day deprived cortex.

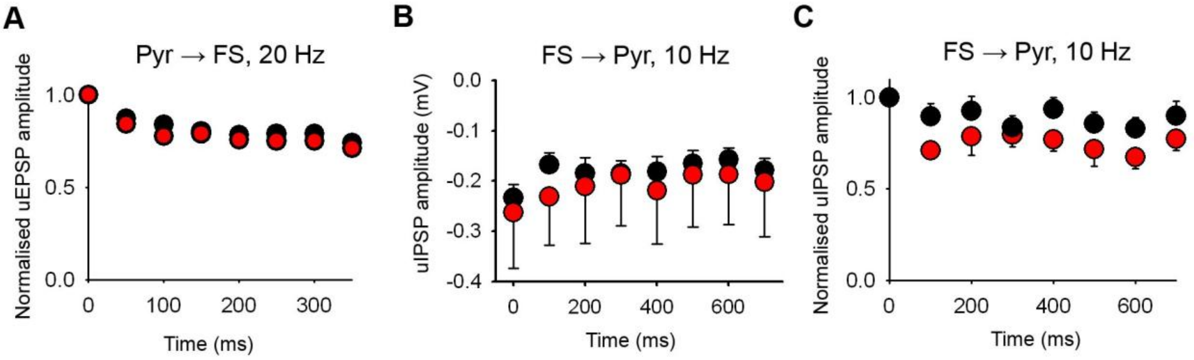

(**A**) uEPSP amplitudes during a 20 Hz train normalized to uEPSP1 for each L2/3 Pyr → FS connection in 3-day deprived cortex (red, n = 25) and in control cortex (black, n = 19). Error bars are within the circles. (**B**) unitary IPSP amplitude during 10 Hz trains in deprived (red, n = 9 FS → Pyr connections) and control (black, n = 6 FS → Pyr connections) cortex. Error bars, s.e.m.. (**C**) uIPSP amplitudes during a 10 Hz train normalized to uIPSP1 for each L2/3 FS → Pyr connection in 3-day deprived cortex (red, n = 9) and in control cortex (black, n = 6). Error bars are within the majority of circles.
